# Green Procedure for Aerobic Oxidation of Benzylic Alcohols with Palladium Supported on Iota-Carrageenan in Ethanol

**DOI:** 10.3390/polym13040498

**Published:** 2021-02-05

**Authors:** Eliraz Stamker, Oshrat Levy-Ontman, Adi Wolfson

**Affiliations:** Department of Chemical Engineering, Sami Shamoon College of Engineering, Basel/Bialik Sts., Beer-Sheva 8410001, Israel; elirast@sce.ac.il

**Keywords:** catalysis, carrageenan, heterogeneous catalysts, oxidation

## Abstract

The search for selective heterogeneous catalysts for the aerobic oxidation of alcohols to ketones and aldehydes has drawn much attention in the last decade. To that end, different palladium-based catalysts have been proposed that use various organic and inorganic supports. In addition, supports that originate from a biological and renewable source that is also nontoxic and biodegradable were found to be superior. We heterogenized palladium chloride or acetate complexes with triphenylphosphine trisulfonate on iota-carrageenan xerogel by simple mixing of the complex and the polysaccharide in water. The resulting polysaccharide-catalyst mixture then underwent deep freeze and lyophilization, after which the catalyst was characterized by TEM, XPS and SEM-EDS and tested in aerobic oxidation. The new heterogeneous catalysts were successfully used for the first time in the aerobic oxidation of benzylic alcohols. Moreover, they were easily removed from the reaction mixture and recycled, yielding an increase in activity with each subsequent reuse. As determined by TEM and XPS, the reduction in palladium and the formation of nanoparticles during the reaction in ethanol yielded more active species and, therefore, higher conversion rates. A SEM-EDS analysis indicated that the palladium was thoroughly dispersed in the xerogel catalysts. Moreover, the xerogel catalyst was observed to undergo a structural change during the reaction. To conclude, the new heterogeneous catalyst was prepared by a simple and straightforward method that used a non-toxic, renewable and biodegradable support to yield an active, selective and recyclable heterogeneous system.

## 1. Introduction

The selective oxidation of alcohols to ketones and aldehydes, which has been attracting significant attention both in laboratory scale studies of catalysis and in industrial production, is considered a pivotal reaction in organic synthesis [1,2,3,4]. However, the scaled-up application of oxidation reactions in synthesis has been severely limited by the large amounts of hazardous metal oxidants required, such as manganese or chromium oxides, which also necessitate the use of toxic solvents such as DMF and DMSO and produce excessive amounts of effluents [5]. Furthermore, running oxidation with these strong oxidants tends to result in over-oxidized products, e.g., primary alcohols are oxidized to their corresponding carboxylic acids.

To avoid the use of metal oxidants and toxic organic solvents, a cleaner and safer oxidation method was developed that uses hydrogen peroxide in water. That method, however, is limited by the low solubility of many organic compounds in water and the fact that water can react with other functional groups on the molecule [6,7]. An alternative is to use aerobic oxidation in the presence of a transition metal-based catalyst. Such a method utilizes low-cost and readily available oxygen as the oxidant while producing only water as a by-product. As such, it constitutes a “green route” for alcohol oxidation that can be performed in variety of organic solvents [8,9].

Over the years, many homogeneous catalysts have been suggested for this purpose, among them systems with copper [10] and iron [11] or transition-metal complexes [12,13] based mainly on palladium or ruthenium [14,15]. Yet if the intention is to develop an environmentally friendly, simple and inexpensive industrial protocol, homogeneous systems suffer a huge drawback: separation of the metal from the reaction mixture at the end of the reaction is a complicated process in such systems, and the catalyst cannot be recycled. In heterogeneous systems, in contrast, where the metal is immobilized on a support, the end-products are easy to separate and recycle. These benefits notwithstanding, the preparation of any heterogeneous catalyst must be as simple and as reproducible as possible, while avoiding any leaching of the metal [16,17].

A variety of supported metals have been offered as heterogeneous catalysts for the oxidation of alcohols [18,19] by using different supports, for example, Pd/Alumina [20], Ni/Hydrotalcite [21], Ru/Hydroxyapatite [22], Co/Activated carbon [23], and Au/Polystyrene [24]. Palladium catalysts, which have found widespread application in organic synthesis, were also extensively used to this end, and they employed different supports, such as silica [25], graphene [26], aluminum hydroxide [27], MnCeOx [28], and poly(ethyleneglycole) [29]. Additionally, the most active species in many systems were palladium nanoparticles [29,30,31,32,33].

Since many organic and inorganic supports can potentially be used, supports that originate from a biological and renewable source that is also nontoxic and biodegradable are superior. To that end, polysaccharides, which constitute the most prevalent form of the biopolymers and the most abundant organic material on earth, have recently been used to immobilize different metal catalysts as complexes or nanoparticles. Of note are the palladium-based catalysts, which have been used mainly in the Suzuki cross-coupling reaction [34,35] but also in other coupling reactions [36], in amination and in hydrogenation [37]. However, polysaccharides-based palladium catalysts for the aerobic oxidation of alcohols have not been proposed. Recently, we also proposed a simple and straightforward procedure to immobilize palladium complexes on various renewable polysaccharides by using sodium triphenylphosphine trisulfonate (TPPTS) as ligand and anchor. The new heterogeneous catalysts were successfully used in Suzuki cross-coupling [38,39,40,41,42] and Heck coupling and in the transfer hydrogenation of olefins [43,44].

**Figure 1:**
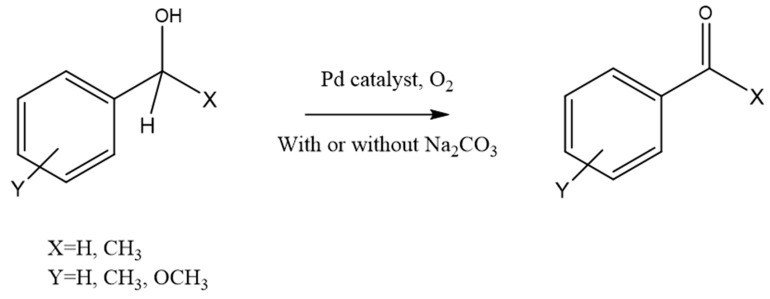
Aerobic oxidation of benzylic alcohols.

In this study, we report for the first time on the use of a palladium catalyst immobilized on a renewable polysaccharide in the aerobic oxidation of benzylic alcohols (Figure 1).

## 2. Materials and Methods

### 2.1. Polysaccharides and Reagents

The polysaccharides, palladium acetate, palladium chloride, TPPTS, benzyl alcohol and sodium carbonate (analytical grades) were purchased from Sigma-Aldrich, Rehovot, Israel.

### 2.2. Catalyst Preparation

In a typical procedure, 10 µmol of palladium salt was added to a vial with 3 mL distilled water together with 30 µmol of TPPTS and mixed at room temperature for 5 min. The mixture was added to a 15-mL polypropylene tube together with 3 mL of 1% wt/vol polysaccharide solution in distilled water, sealed and vortexed for homogenization. The tube was then placed in a deep freeze unit at −20 °C for 24 h until the liquid was completely frozen. Then the seal was removed from the tube and it was covered with a paraffin sheet that was pierced with disposable toothpick. The tube was placed in a lyophilizer (Christ, Osterode, Germany) for 48 h. At the end of the process, the dried xerogel was cut into pieces measuring ~1 cm × 1 cm and added to the reaction mixture.

### 2.3. Reaction Procedure

In a typical procedure, 10 µmol of palladium catalyst (homogenous or heterogeneous) was added to a vial with 5 mL ethanol together with 0.925 mmol benzyl alcohol (S/C = 92.5), with or without 0.092 μmol sodium carbonate. The mixture was placed in an oil bath preheated to 60 °C and magnetically stirred for 24 h. At the end of the reaction, the reaction mixture was cooled, and after reactions in which a heterogeneous catalyst was used, it was removed by filtration through a 0.45 μm Millex LH filter (Millipore, Bedford, MA, USA). The organic phase was then analyzed to determine conversion by GC by using an ZB-5 column (Phenomenex, Torrance, CA, USA). High-pressure reactions were tested in a home-made 10 mL stainless steel reactor with magnetic stirring following the same procedure. 

Catalyst leaching was tested as follows: (1) testing the reaction performance of the catalyst that was removed from the original reaction mixture in a second reaction with a fresh reaction mixture that contains the initial amounts of fresh substrates and sodium carbonate used in the first reaction; (2) testing the reaction performances after catalyst removal by running the reaction mixture under similar conditions for an additional 24 h to test whether the conversion increases with time, and (3) performing spectro arcos ICP-OES (Agilent, Santa Clara, CA, USA) analysis of the reaction medium after the first cycle (24 h), to test for leftover palladium in the reaction solution.

Catalyst recycling was tested by adding the recovered catalyst to a solution with similar amounts of fresh substrates and base and running the reaction mixture under similar reaction conditions for an additional 24 h.

### 2.4. TEM Analysis

HRTEM micrographs were obtained on a EFI Talos F200C electron microscope (Thermo Fischer Scientific, Gloucester, UK) operated at 200 kV at room temperature. The samples were prepared by deposition of a drop of ethanol suspension of the solid catalyst on a carbon-coated Cu grid and examined as grain mounts.

### 2.5. Surface Analysis by X-ray Photoelectron Spectroscopy (XPS)

XPS data were collected by using an X-ray photoelectron spectrometer ESCALAB 250 (Thermo Fischer Scientific, Gloucester, UK) ultrahigh vacuum (1 × 10^−9^ bar) apparatus with an AlK^α^ X-ray source and a monochromator. The X-ray beam size was 500 μm, and survey spectra were recorded with a pass energy (PE) of 150 eV, and high energy resolution spectra were recorded with a pass energy (PE) of 20 eV. To correct for charging effects, all spectra were calibrated relative to a carbon C 1s peak positioned at 284.8 eV. Processing of the XPS results was carried out by using AVANTAGE program (Thermo Fischer Scientific, Gloucester, UK).

### 2.6. Scanning Electron Microscope (SEM) Energy Dispersive X-ray Spectrometry (EDS)

Elemental analysis was performed by using a scanning electron microscope (SEM), FEI Verios 460L XHR (extreme high resolution, Hillsboro, OR, USA), equipped with energy-dispersive X-ray spectroscopy (Thermo Fischer Scientific, Gloucester, UK).

### 2.7. Analysis of Total Sugar Concentration

At the end of the reaction, the catalyst was separated from the reaction solution and washed with ethanol, which was added to the ethanolic reaction solution, and the mixture was then filtered through a 0.45-μm Millex LH filter. The filtrate was dried by speed-vac, and the residue was dissolved in 1 mL of distilled water. The total sugar concentration in the solution was analyzed by phenol-sulfuric assay [45]. Briefly, 1 mL of sample was mixed with 1 mL of 5% phenol and 5 mL of 98% sulfuric acid and incubated at room temperature for about 1 h. Absorbance of the developed color was determined spectrophotometrically at 490 nm. The amount of sugar content was deduced from the absorbance by comparing it with a standard curve (0–100 μg/mL) of galactose.

## 3. Results and Discussion

The investigation began with the homogeneous aerobic oxidation of benzyl alcohol in ethanol—an inexpensive, commercially available and relatively nontoxic and non-hazardous solvent—in the presence of various palladium salt catalysts (Table 1). In all of the reactions, only benzaldehyde was detected, without any benzoic acid or benzyl acetate leftovers, showing that the reaction is selective.

First, two commercially available palladium salts, palladium acetate and palladium chloride, were employed with or without the addition of a base: sodium carbonate (Table 1, entries 1 and 2). As can be seen from Table 1, the conversion rate with Pd(OAc)_2_ was found to be higher than with PdCl_2_ (Table 1, entry 1). Furthermore, while adding sodium carbonate to Pd(OAc)_2_ decreased the conversion rate, it increased the conversion rate when added to PdCl_2_, which was inactive without a base. Here the base assists both with the abstraction of the acidic alcohol of the benzyl alcohol, thus initiating the reaction, and with the dissociation of the anion from the catalyst.

Additionally, the addition of triphenyl phosphine (TPP) as a ligand, which can stabilize the catalyst and prevent reduction in the metal and formation of palladium black, resulted in lower conversion rates with Pd(OAc)_2_, but higher conversion rates with PdCl_2_ in the presence of a base (Table 1, entries 3 and 4). These results emphasize the difference between the two palladium salts, which can be attributed to their different solubilities in ethanol and the different natures of the chloride and acetate ions. Finally, as the heterogenization procedure involves the use of TPPTS as a ligand, the homogeneous reactions with both Pd(OAc)_2_(TPPTS)_2_ and PdCl_2_(TPPTS)_2_ were also tested (Table 1, entries 5 and 6). In the latter case, the conversion rate with Pd(OAc)_2_(TPPTS)_2_ was lower than that with the free salt and higher than Pd(OAc)_2_(TPP)_2_, but still higher than the conversion rate with PdCl_2_(TPPTS)_2_, which was similar to that when using salt alone.

Based on our previous work, iota (*i*) carrageenan was selected as the support for the heterogeneous system [38,39,40,41,42,43,44]. With this support, the reaction with *i*-Pd(OAc)_2_(TPPTS)_2_ was superior to that with *i*-PdCl_2_(TPPTS)_2_ (Table 1, entries 7 and 8). Increasing the air pressure from 1 atm to 3.8 atm slightly decreased the conversion rates, perhaps because of the high concentration of nitrogen that was adsorbed on the palladium surface (Table 1, entries 9 and 10). Lastly, various benzylic alcohols were tested and also successfully oxidized using the new heterogeneous catalysts. In those tests, the addition of an electron donating group, such as methoxy, was found to significantly increase the conversion rate (Table 1, entries 11–13). However, aliphatic alcohols, which are less prone to aerobic oxidation, showed only negligible conversion rates. Next, to better evaluate the applicability of the new heterogeneous system, we tested the ability of both *i*-based catalysts to be recycled and the ability of the system to function for multiple runs (Table 2).

As illustrated in Table 2, in the new heterogeneous systems the catalysts could be easily separated from the reaction mixture and recycled, while running the reaction again with the filtered reaction mixture after catalyst removal did not result in an increase in the conversion, thus hinting that the catalyst had not leached into the solution. Furthermore, ICP-OES analysis did not reveal any leftover palladium in the solution, indicating that there was no palladium leaching. Moreover, and surprisingly, the reaction cycles run after the first and second times the catalysts were recycled (Table 2, entries 2 and 3) yielded higher conversion rates than the initial reaction cycle. To test the stability of the heterogeneous catalyst under the reaction conditions, the sugar content in the reaction mixture after each cycle was measured by using the colorimetric phenol-sulphuric method. The sugar content in the reaction solution at the end of each cycle was negligible compared to the amount of polysaccharide used to prepare the catalyst (less than 50 µg sugar compared to 0.03 g polysaccharide initially used). This observation indicates that the heterogeneous catalyst almost did not degrade and/or was dissolved in the reaction solution, even though the reaction conditions comprised mixing at under 60 °C for 72 h.

In addition, the color of the catalyst grew darker from cycle to cycle. We suggest that this may be attributed to the formation of palladium nanoparticles while heating the catalyst under reaction conditions, as was previously reported under similar reaction conditions in a Suzuki-cross coupling in ethanol [35].

**Figure 2:**
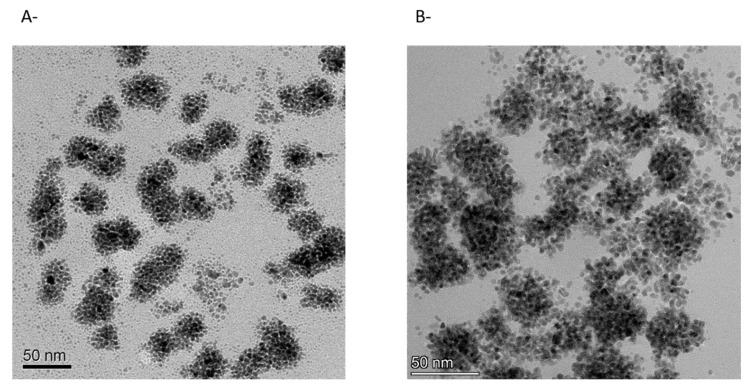
TEM micrographs of *i*-Pd(OAc)_2_(TPPTS)_2_: (**A**) Before reaction; (**B**) After 24 h of reaction.

The next step was to rigorously evaluate the state and form of the palladium (and its distribution within the fresh heterogeneous catalyst) and the catalyst after 24 h of reaction. Both samples were analyzed by TEM (Figure 2).

As expected, the TEM image of *i*-Pd(OAc)_2_(TPPTS)_2_ fresh catalyst (Figure 2A) showed that nanoparticles had already been created during the lyophilization of the polysaccharide-catalyst mixture, which is in agreement with previously published findings [38,39]. However, employing the catalysts in the reaction and heating it for 24 h at 60 °C resulted in the formation of much larger nanoparticles than previously reported, as illustrated in Figure 2B. This implies that the reaction conditions promoted reduction in the palladium and nanoparticle aggregation [39].

Nanoparticles of palladium have been intensively studied in a wide range of catalytic applications, including C-C coupling reactions (e.g., Suzuki, Heck, Sonogashira, Negishi, Stille, Kumada and Hiyama), hydrogenation and electrochemical reactions in fuel cells [33,35]. There are also several reports showing the successful employment of palladium nanoparticles in oxidation reactions. For example, palladium nanocatalysts with carbon nanomaterial support have been successfully applied in a glucose oxidation reaction [46]. Additionally, a biphasic aerobic oxidation of alcohols was performed by using palladium nanoparticles in a polyethylene glycol matrix as the catalyst and supercritical carbon dioxide as the substrate and product phase [29]. In contrast to our findings, however, it is well known that the aggregation of palladium nanoparticles, which results in a reduction in the surface area-to volume ratio, usually renders palladium nanoparticles less active and selective [33]. It was therefore suggested that other factors are responsible for the reaction enhancement effect, such as changes in the palladium oxidation state, palladium distribution on the surface, palladium nanoparticle morphologies, rate of nanoparticle formation, changes in the support structure, mass transfer limitations, etc.

Analysis by XPS was used to better understand the changes in the elemental distribution on the heterogeneous catalyst and to determine the oxidation state of the palladium on the surface before and after the reaction. The analysis showed that the palladium atomic concentration on the surface of both preparations was similar, 0.5%, and in agreement with the amount that was used to form the heterogeneous catalyst. Moreover, the elemental identification and quantification of *i*-Pd(OAc)_2_(TPPTS)_2_ before and after the reaction was also similar in both preparations, as shown in Table 3.

**Figure 3:**
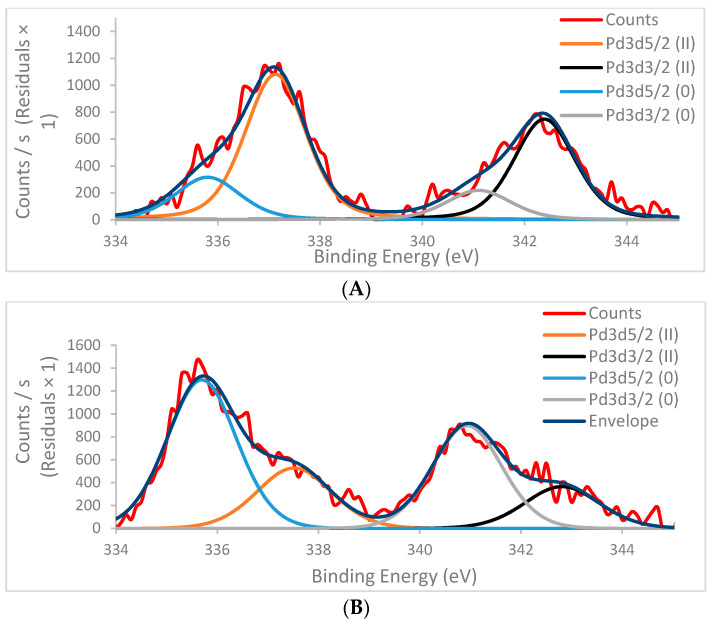
XPS spectra of *i*-Pd(OAc)_2_(TPPTS)_2_ in the Pd3d region (**A**) Before reaction, (**B**) After 24 h of reaction.

Curve-fitting of the Pd3d spectra of the two preparations (Figure 3) shows that both consist of a doublet (Pd3d5/2 and Pd3d3/2) due to spin-orbit splitting, and each component of the doublet involves two peaks, assigned to Pd(0) and Pd(II). However, the two samples exhibited different ratios between the two oxidation states.

In the spectrum results for the fresh heterogeneous catalyst, the area percentage peaks of the Pd(0) and Pd(II) correspond to 24% and 76%, respectively (Figure 3A). For the post-reaction heterogeneous catalyst, the area percentage peaks of the Pd(0) and Pd(II) correspond to 70% and 30%, respectively (Figure 3B). This shows that 46% of the Pd(II), derived from Pd(OAc)_2_, was reduced to metallic form during 24 h of reaction with binding energies of Pd(0) 3d (d5 335.6 eV; d3 341.6 eV) (Figure 3). Thus, it seems that the enhancement in catalytic activity after recycling of the xerogel can be attributed to the increase in the Pd(0) form.

**Figure 4:**
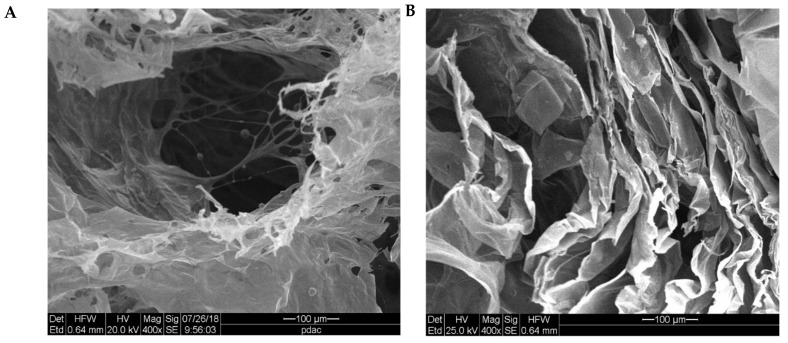
Secondary scanning electron microscopy (SEM) imaging of *i*-Pd(OAc)_2_(TPPTS)_2_: (**A**) Before reaction; (**B**) After 24 h of reaction.

The elemental analysis of *i*-Pd(OAc)_2_(TPPTS)_2_ before and after the reaction was also investigated via SEM-EDS (Figure 4). Representative EDS spectra of various samples revealed that the palladium atomic concentration in both preparations was 0.5%, which is in agreement with the amount used to form the xerogel catalyst and the amount shown in the XPS analysis, indicating that the palladium was probably uniformly dispersed in the xerogel. Similarly, the molar elemental ratio of P:Pd in the heterogeneous catalyst was found to be 3:1, as in the homogeneous catalyst (data not shown). What this suggests is that the complex, *i*-Pd(OAc)_2_(TPPTS)_2_, has kept its elemental composition despite the reaction conditions. However, the images of both samples obtained using SEM indicate that the structures are different. While the *i*-Pd(OAc)_2_(TPPTS)_2_ xerogel before the reaction is characterized by a porous sphere or structure with hollows (Figure 4A), the xerogel that appears during the 24 h of reaction is characterized by ordered vertical porous tubes (Figure 4B). Indeed, the structural differences can be observed by physical contact: the xerogel becomes more rigid after the reaction. Therefore, it seems that structural changes also play a major factor that can pose a mass transfer limitation that can affect reaction performances between successive cycles.

## 4. Conclusions

*i*-Pd(OAc)_2_(TPPTS)_2_ and *i*-PdCl_2_(TPPTS)_2_, prepared using a very simple and straightforward method, were successfully employed in the aerobic oxidation of benzylic alcohols. After 24 h pf reaction, the conversion rate of benzyl alcohol with *i*-Pd(OAc)_2_(TPPTS)_2_ (22%) was higher than that with *i*-PdCl_2_(TPPTS)_2_ (8%) and close to that of the parent homogeneous complex (32%). In this novel method, the metal did not leach into the reaction mixture and the catalyst was successfully recycled twice, exhibiting increases in activity in the reactions after the first and second times it was recycled from an initial conversion rate of 22% to 29% in the first recycle and 35% in the second. Notably, both the catalyst matrix and the solvent used in this approach are environmentally preferable, thus making this approach of general interest for the pursuit of viable “green” nanoparticle catalysis systems. Characterization of the lyophilized *i*- Pd(OAc)_2_(TPPTS)_2_ system via TEM, XPS and SEM-EDS analyses before and after the reaction in ethanol shows that the palladium was successfully dispersed and embedded in the xerogel and that palladium nanoparticles formed during the reaction and led to higher conversion rates. In addition, the structure of the *i*-Pd(OAc)_2_(TPPTS)_2_ was altered in the course of the reaction.

## Figures and Tables

**Table 1:** Homogeneous and heterogeneous reactions in ethanol ^a^.

Entry	Catalyst	Base ^b^	Pd(OAc)_2_ Conversion (%)	PdCl_2_ Conversion (%)
1	Homogeneous-No ligand	-	50	0
2	Homogeneous-No ligand	+	37	19
3	Homogeneous-TPP	-	23	0
4	Homogeneous-TPP	+	28	30
5	Homogeneous-TPPTS	-	39	0
6	Homogeneous-TPPTS	+	32	20
7	Heterogeneous	-	20	0
8	Heterogeneous	+	22	8
9	Heterogeneous ^c^	-	12	0
10	Heterogeneous ^c^	+	20	12
11	Heterogeneous ^d^	-	21	14
12	Heterogeneous ^e^	-	22	not tested
13	Heterogeneous ^f^	-	40	not tested

^a^ Reaction conditions: 0.925 mmol benzyl alcohol, 10 µmol catalyst, 5 mL solvent, 60 °C, 1 atm air, 24 h. ^b^ + refers to an addition of 0.092 µmol Na_2_CO_3; -_ without basis. ^c^ 3.8 atm air. ^d^ 1-Phenylethanol instead of benzyl alcohol. ^e^ 4-Methylbenzyl alcohol instead of benzyl alcohol. ^f^ 4-Methoxybenzyl alcohol instead of benzyl alcohol.

**Table 2:** Catalyst recycling in ethanol ^a^.

Cycle	*i*-Pd(OAc)_2_(TPPTS)_2_ Conversion (%)	*i*-PdCl_2_(TPPTS)_2_ Conversion (%) ^b^
1	20	8
2	29	11
3	35	15

^a^ Reaction conditions: 0.925 mmol benzyl alcohol, 10 µmol catalyst, 5 mL solvent, 60 °C, 1 atm air, 24 h. ^b^ Addition of 0.092 µmol Na_2_CO_3_.

**Table 3:** Elemental identification and quantification of *i*-Pd(OAc)_2_(TPPTS)_2_ before and after reaction, determined by XPS.

Element	Atomic % before Reaction	Atomic % before Reaction
C1s	56.76	55.99
O1s	31.91	31.26
N1s	0.58	0.51
Pd3d	0.50	0.49
Na1s	2.07	2.75
Ca2p	1.09	1.32
S2p	6.43	6.98
K2p	0.68	0.69

## Data Availability

The data presented in this study are available on request from the corresponding author.
